# Case of Prostatic Abscess

**Published:** 1852-11

**Authors:** James P. White

**Affiliations:** Buffalo, N. Y.


### ART. V. — *Case of Prostatic Abscess.* Reported by Prof. James P. White, Buffalo, N. Y.

Below you will find a brief account of a case of acute inflammation terminating in abscess of the prostate gland, with the course of treatment pursued. It interested me by reason of the ambiguity of the symptoms and its rarity during its progress; and may not be entirely without interest to some of the readers of your valuable Journal.

On the 7th of September last, visited for the first time Mr. B., a gentleman, forty-eight years of age, of sedentary habits, but of general good health. He had suffered from irritation of the urethra or neck of the bladder, during the last two years, and had been treated by a surgeon in New York, for stricture. He had been supplied with bougies, of graduated sizes, and directed to insert them occasionally to prevent contraction. During this period he had passed a large bougie every fifteen or twenty days without difficulty, but it was always attended with great irritation and pain throughout the entire canal. The insertion of the instrument gave rise to considerable pain in the glans penis also. He had introduced one of these large bougies quite to the bladder, about a week before I saw him, which gave him more than the ordinary amount of pain at the time, and was succeeded by an unusual degree of suffering during micturition. Subsequently he had, however, traveled several hundred miles by railroad, and indulged more freely than usual in stimulating food and drinks. He had also suffered frequently from the descent of hæmorrhoidal tumors, one of which, as large as an almond, was now in an inflamed condition, attached to the posterior margin of the anus. The pulse was considerably accelerated; there was a general febrile movement with frequent micturition and tenesmus. The passage of the urine occasioned an acute burning sensation in the urethra, although external pressure did not produce pain. The urine was nearly natural in appearance, there being only a slight mucous deposit after standing.

Directed the patient to preserve the horizontal posture, applied cold to the hæmorrhoidal tumor, giving him freely of Dover's powder and morphine. He also took a teaspoon, three or four times each day, of a mixture of spirits of nitre and the muriated tinct. of iron. Under this treatment the fever subsided, and the acuteness of the pain was somewhat relieved for a day or two. On the 11th, however, the pains were increased in severity, and the patient found it exceedingly difficult to locate them. The whole perimeal region seemed to participate in the painful sensations, though pressure upon this part did not increase it. These pains were intermitting, increased in the after part of the day, and accompanied by a sense of weight in the pelvis. Apprehensive that the symptoms were of too grave a character to be entirely accounted for by the presence of piles, and doubting the existence of any considerable contraction of the urethra, a rectal examination was insisted upon. The finger being carried well up into the rectum, immediately came in contact with a hard tumor upon the right side of the neck of the bladder. This was, doubtless, the right lobe of the prostate gland. It was exceedingly sensitive to the touch, and as large as a Madeira-nut.

Continued the horizontal position, applied a dozen leeches to the perineum, and followed with warm anodyne fomentations. Gave also laudanum or morphine, by enema, in such quantities as to moderate the sufferings of the patient. The bowels were moved every second day with castor oil, and, it should be added, that defecation was attended with severe pain.

On the following day Dr. Austin Flint saw the patient, in consultation with myself, and concurred in the diagnosis and in the treatment which was being pursued. Continued the discutient treatment until it became evident by a repetition of the rectal examination that suppuration had commenced.

On the 17th, in order to verify the diagnosis, in the presence of Prof. Flint, carried an exploring canula and needle up beside the finger, and pushed it into the soft tumor, upon which the finger rested. A few drops of well-digested pus immediately followed the withdrawal of the stilet, and demonstrated the nature of its contents. The tenseness of the tumor was diminished and the pain became less severe, after this slight discharge of matter.

On the following day, September 18th, after evacuating the bowels, a small, long trochar was carried up upon the finger, and after placing it in contact with the sac, the stilet was thrust through its parieties, when it was immediately withdrawn and the canula pushed forward into its cavity. There now escaped something less than a gill of pure pus through the instrument, when a syringe was nicely fitted to the outer extremity, and suction applied, in order to effect its complete evacuation. The soft tumor immediately collapsed, and great relief to all the urgent symptoms followed. The bowels were left unmoved for several days, in order, if possible, to permit adhesion between the parieties of the abscess, and prevent the continued formation of pus and a fistulous opening into the rectum. The patient improved rapidly without the discharge of any more purulent matter, and an examination one month after the operation, enables me to detect the prostate somewhat enlarged, but not painful on pressure, and no trace of the orifice through which the pus was withdrawn to be detected. He has resumed his ordinary pursuits and has less pain and fewer of the symptoms which he attributed to stricture, than have been present at any time during the last two years. It may be added that he is, however, under treatment for the discussion of the chronic or senile enlargement which I believe gave rise to the symptoms attributed to stricture, and which, I doubt not, is diminished by the suppuration which has just terminated.

This acute form of disease in the prostate is rare, and, so far as my reading extends, the course to be pursued when matter is formed, not well established. I am not aware that the method pursued in this instance has ever before been resorted to. Most standard works on surgery omit to mention the existence of this affection. Civiale and Gross, the best authorities in the French and English languages, recommend that, when detected, exit should be given to the purulent collection through the perineum, by puncture. Now this would be difficult to accomplish, on account of the amount of tissue to be divided, and would almost certainly end in fistula. Many authors insist that abscess of the prostate shall be left to rupture spontaneously. It is at the same time conceded that rupture into the peritoneal cavity would be certainly fatal; and that a connection (which is most likely to be established) between the abscess and the bladder would be scarcely less to be dreaded. Could we be sure that the matter would be discharged through the perineum, the demand for surgical interference would be less imperative. But the distance of the gland from this surface and the deneness of the tissues through which the matter must pass, render this result highly improbable. Again, should the abscess open into the rectum, the only other probable outlet, if left to rupture itself, it would unquestionably leave a large open sac to be filled with feculent matter, and result in a very bad form of fistulous connection. In view of all the circumstances it would seem very desirable that the fluid should be artificially drawn off at as early a period as practicable after its discovery, and prevent, if possible, any of the fearful consequences so likely to result from spontaneous rupture.

October, 1852.

